# MR findings of primary ovarian granulosa cell tumor with focus on the differentiation with other ovarian sex cord-stromal tumors

**DOI:** 10.1186/s13048-018-0416-x

**Published:** 2018-06-05

**Authors:** He Zhang, Hongyu Zhang, Shouxin Gu, Yanyu Zhang, Xuefen Liu, Guofu Zhang

**Affiliations:** 1 Department of Radiology, Obstetrics and Gynecology Hospital, Fudan University, Shanghai, 200011 People’s Republic of China; 2 Department of Ultrasound, Daqing Oilfield General Hospital, Daqing, Heilongjiang Province 163001 People’s Republic of China; 3 Institute of functional and molecular medical imaging, Fudan University, Shanghai, 200040 People’s Republic of China

**Keywords:** Ovarian granulosa cell tumor, Sex-cord tumor, MRI, Diagnostic imaging

## Abstract

**Background:**

To describe magnetic resonance imaging (MRI) features of ovarian granulosa cell tumors (OGCTs) and compare with other sex cord-stromal tumors (OSCs) in ovary.

**Methods:**

MR findings of 18 patients with surgically confirmed ovarian granulosa cell tumor were retrospectively reviewed by two radiologists with consensus reading. All MR examinations were prospectively performed within one month. Clinical and imaging characteristics of OGCTs were evaluated and compared with OSCs (control group).

**Results:**

In 18 patients, 20 ovarian granulosa cell tumors were detected on MRI. Sixteen tumors appeared as solid or mostly solid mass (16/20), while 4 tumors as cystic mass. Pathological pelvic fluid was detected in 1 OGCT (1/18) and 11 OSCs (11/34) (*p* = 0.031).On T2 weighted imaging (T2WI), most of OGCTs displayed hyperintense signal and mixed signal (19/20); on T1 weighted imaging (T1WI), 11 OGCTs (11/20) displayed similar signal as on T2WI imaging. The lesion signal between OGCT and OSC differed significantly on both T1WI (*p* = 0.017) and T2WI (*p* = 0.002). Tumoral bleeding was detected in 6 OGCTs on MRI. On diffusion weighted imaging (DWI) images, OGCTs mostly appeared as high signal (16/20). Average apparent diffusion coefficient (ADC) value derived from DWI images in the OGCT group (0.84 ± 0.26× 10^− 3^ mm^2^/s was less than the control group (1.22 ± 0.47 × 10^− 3^ mm^2^/s) with statistical difference (*p* = 0.002).

**Conclusions:**

MRI could provide important information in OGCT diagnosis. ADC value might be useful in differentiating OGCT from OSC.

## Background

Ovarian granulosa cell tumor (OGCT) is a rare sex cord-stromal tumor in ovary, accounting only 2–3% of all ovarian tumors [1]. Pathologically, OGCTs are classified into two subtypes: adult and juvenile form, in which adult type occupying 95% of all OGCTs [2]. Despite OGCT have a favorable prognosis, an incidence of 25–30% metastases or recurrences make it as a low malignant potential ovarian tumor [3]. Chemotherapy is recommended as adjuvant treatment for patients with stages II–IV granulosa cell tumor [4–7]. Owing to the superb soft-tissue resolution and free radiation, magnetic resonance imaging (MRI) is widely used as a problem-solving modality in assessment of complex adnexal masses that are indeterminate on ultrasonography (US) or computed tomography (CT) [8]. Till now, most of reported OGCTs in the literatures are published as case report and no detailed MRI knowledges of OGCTs have been comprehensively described [6–13]. In this study, by evaluating OGCTs in our single institution, we aimed to: (1) thoroughly evaluate the MRI appearances of OGCTs in a large cohort of samples and record ADC values for each lesion; (2) compare these features with OSCs.

## Methods

### Study subjects

Between December 2009 and December 2015, 1217 consecutive patients with clinically suspected adnexal disease prospectively underwent 1.5 T MRI examinations before pelvic or laparoscopic surgery at our institution. The time interval between the MRI evaluation and surgery was less than one month (2–27 days; mean, 5 ± 12 days).

Among them, 18 patients with histologically proven OGCT (24–79 years of age; average age, 45.9 ± 15.3 years) were included in this study when we retrospectively retrieved the database on the Picture Archiving and Communication System (PACS). Two recurrent OGCTs were excluded for further analysis because the primary imaging data was evaluated in another hospital. Thirty four patients with OSCs, including histologically proven sclerosing stroma tumors (SST, *n* = 4), fibrothecomas (*n* = 21), and fibromas (*n* = 9), were included as the comparative group. Patients with any previous pelvic surgery or radiation history were arbitrarily excluded because the inherent structure of the uterus may has been altered. Details of the samples studied are summarized in Table 1.

**Table 1:** Summaries of histological results in 54 ovarian sex-cord lesions detected on MRI in 52 patients

Pathology diagnosis	Numbers
Granulosa cell tumor	20^a^
Fibrothecoma	21
Sclerosing stroma tumor	4
Fibroma	9

^a^indicates 20 lesions in 18 patients

### Image acquisition

MRI was performed using a 1.5-T MR system (Magnetom Avanto, Siemens, Erlangen, Germany) with a phased-array coil. The routine MRI protocols used for assessment of pelvic masses included axial turbo spin-echo (TSE) T1WI, sagittal TSE T2WI, and axial/sagittal TSE fat-suppressed T2WI (FS T2WI). For axial images, the transverse plane was perpendicular to the long axis of uterine body; for sagittal images, the longitudinal plane was parallel to the main body of uterus. DWI using an echo-planar imaging two-dimensional (EP2D) sequence performed in the axial plane with parallel acquisition technique by using *b* value = 0, 100, and 800 s/mm^2^. Contrast-enhanced pelvic imaging was acquired at multiple phases of contrast medium enhancement in both sagittal and axial planes.

### Image analysis

The location, size (the largest dimension in two orthogonal planes), margin (regular or irregular); visibility of hemorrhagic component (high signal on T1WI) within the lesion; and presence of capsule, pelvic-free fluid and lymph node were also noted. On T1WI, hypo-, iso-, and hyperintensity were similar for the pelvic fluid, pelvic wall muscle, and fat signal; on T2WI, hypo-, iso-, and hyperintensity were similar for the pelvic bone, pelvic wall muscle, and fat signal; on *b* = 800 mm^− 2^/s DWI images, the low, intermediate, and high-signal intensity were similar for the pelvic bone, myometrium, and endometrium. After the intravenous injection of the contrast medium, the lesion enhancement type was graded as follows: 1, minor enhancement (clearly less than the myometrium); 2, mild enhancement (less than the myometrium); 3, moderate enhancement (similar to the myometrium); or 4, avid enhancement (more than the myometrium). ADCs were measured manually on post-processing workstation (Leonardo, Siemens, Germany) by one reviewer (H.Z.). Two observers (S. X. G. and H.Z., with 6 and 10 years of experience in gynecological imaging, respectively), who were blinded to the histological results independently, analyzed MRI datasets of each participant. At the end of the study, two observers were also required to determine the tumor etiology (benign or malignant) according to previous established criteria [14–16]. For interobserver discrepancies in the evaluation of uterine lesions, consensus was achieved.

### Statistical analyses

Continuous variables were expressed as the means ± standard deviation (S.D.) and compared with the unpaired *t* test if normally distributed or the Mann–Whitney test if not normally distributed. A nonparametric test (Mann–Whitney) was used to test other nonparametric variables within each group. The area under the receiver operating characteristic (ROC) curve (AUC) was calculated for ADCs to discriminate OGCTs from OSCs. SPSS (version 13.0, SPSS, Chicago, USA) was used to perform statistical analyses. *P* values ≤0.05 were considered statistically significant.

## Results

**Fig. 1:**
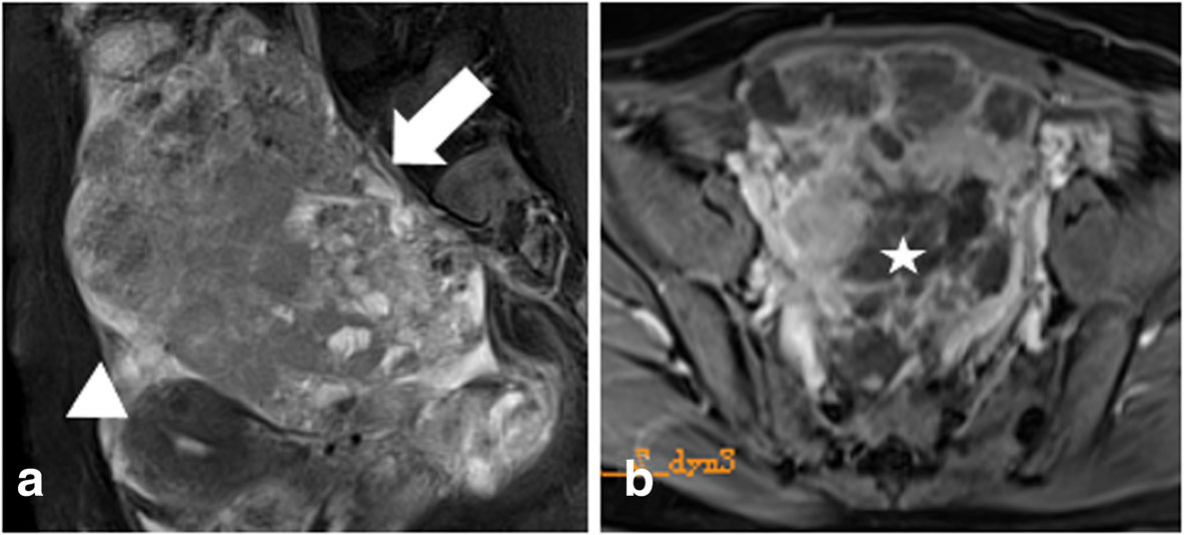
A 32-year-old woman histologically proved OGCT in juvenile type (IIIc). On sagittal FS-T2WI image (**a**), the giant mass with irregular margin (arrow) occupy the majority of pelvis with the uterus being pushed forwardly (arrow head). (**b**) On contrast-enhanced images, the mass show homogeneously moderate enhancement; the necrotic area do not show enhancement (star)

**Fig. 2:**
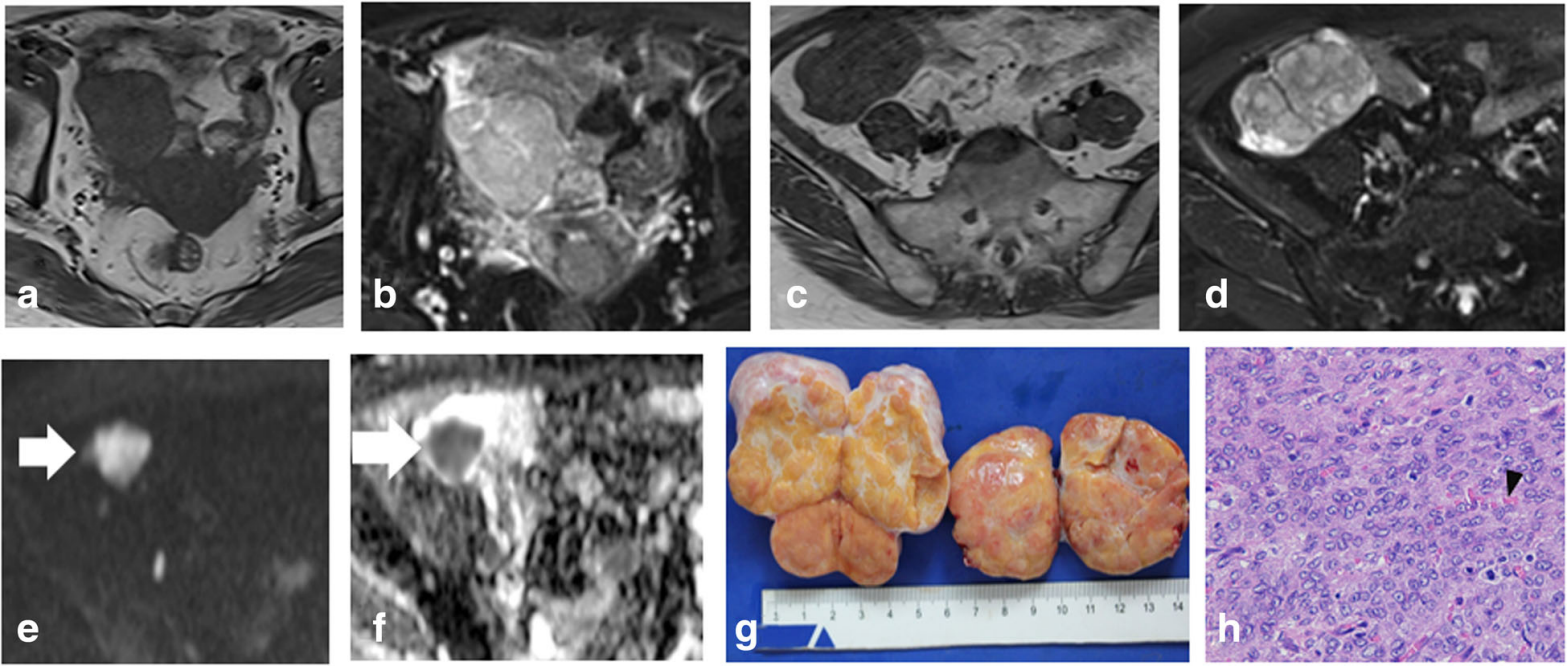
A 61-year-old woman with primary adult type OGCT (Ia). There are oval, solid masses on right adnexal region (**a**, **b**) and right iliac fossa (**c**, **d**). Two lesions appears as similar signals with intermediate signal on both T1WI (**a**, **c**) and FS-T2WI (**b**, **d**) with intact capsule. On DWI image (**e**), the mass at the right iliac fossa shows relatively high signal (arrow) and low signal on the ADC map (**f**). The gross specimen reveals the yellow, solid mass with smooth capsule and a thin septa (**g**). Photomicrograph, hematoxylin and eosin stained section (× 40) shows the oval cells arrange closely with pleomorphic nuclei, prominent nucleoli and scanty cytoplasm. Note, the small vessels embed among the intercellular space (arrow head)

The histological results revealed 20 OGCTs in 18 patients (24–79 years of age; average age, 48.2 ± 15.1 years), including 17 adult types and 1 juvenile type (Fig. 1). Laparotomy was performed in 13 patients while others with laparoscopic surgery. Nine patients had regular or irregular menses, while menopause in nine patients. According to the international federation of obstetrics and gynecology (FIGO) staging system [17], twelve patients were classified as Ia, 4 as Ic, 1 as IIIa and 1 as IIIc. All primary tumors were solitary lesion detected on MRI, except for three primary lesions in one patient at initial evaluation (Fig. 2). Most of OGCTs at presentation appeared as the large mass with the average diameter of 9.33 ± 5.43 mm. Among them, five patients were also accompanied by other ovarian etiologies, including endometrial polyps (*n* = 2), uterine fibroids (*n* = 2), follicular cyst (*n* = 1), Brenner tumor (*n* = 1) and fibroid and mucinous cystadenoma (*n* = 1). Vaginal discharge was recorded in one OG patient. The details of baseline characteristics for all studied samples are summarized in Table 2.

**Table 2:** Basic and imaging characteristics of OGCTs

	OGCTs	OSCs	*P* value	OR^a^
Imaging findings				
T1 signals	20	34	0.017	
hypointensity	4	8		
isointensity	5	26		
hyperintensity	5			
mixed	6			
T2 signals			0.002	
hypointensity		5		
isointensity	1	12		
hyperintensity	12	10		
mixed	7	7		
DWI signals			0.003	
hypointensity	1	7		
isointensity	3	11		
hyperintensity	16	16		
mixed				
Margin			0.326	0.172
Regular/Iregular	17/3	33/1		
Capsule			0.015	0.800
Present/Absent	16/4	34/0		
Hemorrhage			0.001	3.429
Present/Absent	6/14	0/34		
Component			0.000	2.50
Solid(80–100% solid component)	8	28		
Cyst (80–100% cystic component)	4	1		
Solid with cystic changes (others)	8	5		
Enhancement (homogeneous/inhomogeneous)			0.034	
minor		16(16/0)		
mild	14(12/2)	10(7/3)		
moderate	6(5/1)	4(4/0)		
avid		4(1/3)		
Maximum diameter (mm)	9.33 ± 5.43	8.5 ± 8.9	0.753	
< 5	6	17		
5–10	9	12		
> 10	5	5		
ADC values(×10^−3^ s/m^2^)	817 ± 144 (558–1120)	1223 ± 473 (460–2230)	0.002	
Septa			0.121	
Present/Absent	6/14	5/29		
Lymph node			0.913	
Present/Absent	0/18	0/34		
Pelvic fluid			0.031	8.130
Physiological/ Pathological	17/1	23/11		
Clinical findings				
Age(years)	48.2 ± 15.1(24–79)	54.8 ± 15.0(21–81)	0.139	
Vaginal discharge/bleeding	1	0	0.106	
Menstruation				
Regular(irregular)	9(4)	18(5)	0.607	
Menopause	9	16		
Accompanying lesions			0.280	
Yes/ No	7/12	9/25		

^a^Indicates odds ratio

### MRI characteristics

In this studied samples, OGCTs showed varying signal intensities on both T1WI and T2WI images. On T1WI, OGCTs showed various signal from low to mixed signal, which was different to OSCs mostly appearing as hypointense and isointense mass (*p* = 0.017). On T2WI, OGCTs mainly displayed as high and mixed signal (19/20). Accordingly, OSCs mainly displayed as isointense (12/34) and hyperintense signal intensity (10/34) on T2WI images.

**Fig. 3:**
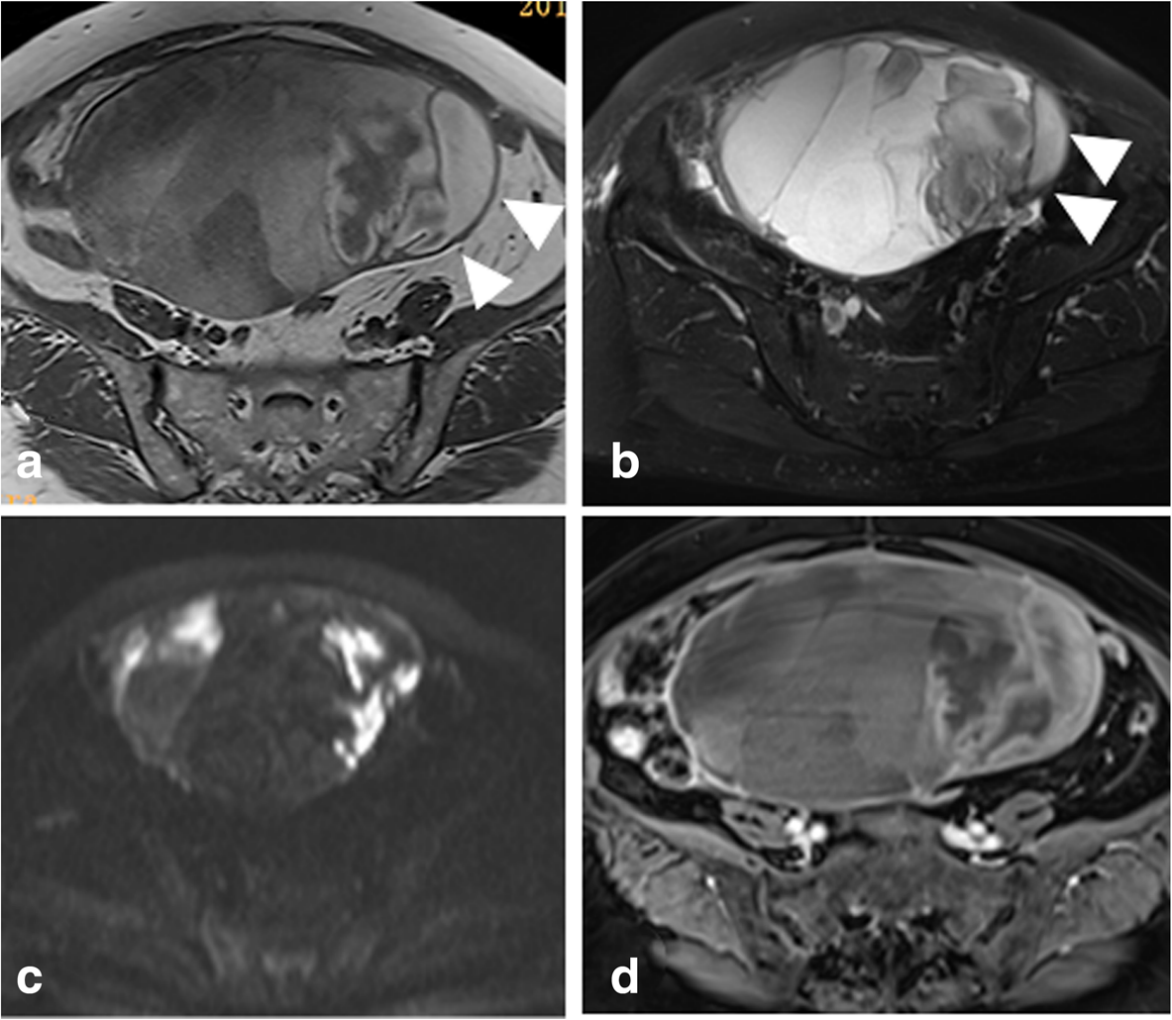
A 54 -year-old woman with primary adult type OGCT (Ic). The mass shows as the purely cystic lesion with mostly high signal on T1WI (**a**) and T2WI (**b**). Note, the hemorrhagic contents locates on the left side of the tumor, representing the relatively high signal on T1WI and low signal on T2WI (arrowhead) and high signal on DWI (**c**). After injection of contrast medium, the cystic wall shows minor enhancement (**d**)

**Fig. 4:**
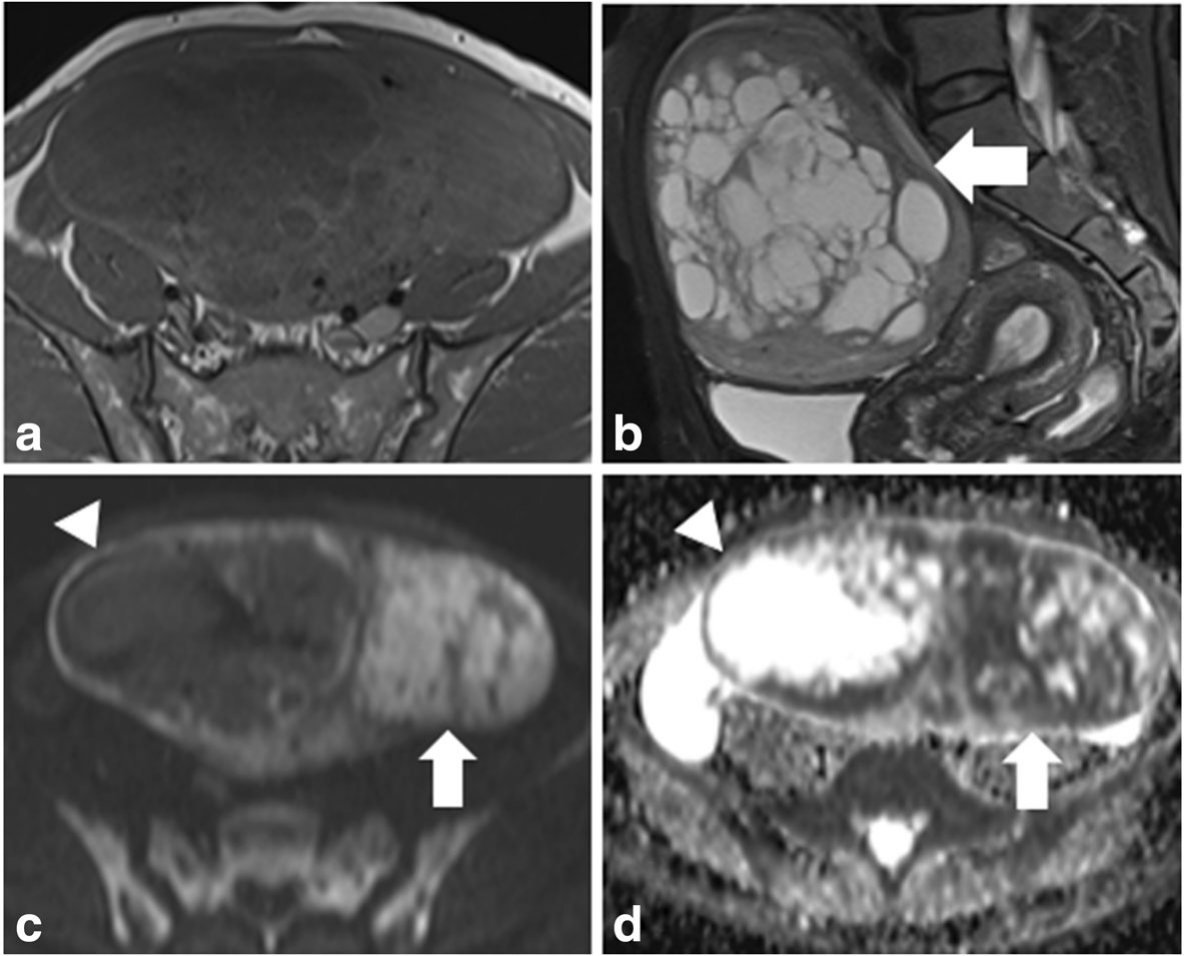
A 50-year-old woman with primary adult type OGCT (IIIa). (**a**) The spongelike changes (arrowhead) are observed in the interior of the tumor appearing as the mostly solid mass (**b**). The cystic contents (arrowhead) give the low signal on DWI (**c**) and relatively high signal on ADC map (**d**), while the solid part (arrow) with the relatively high signal on DWI and low signal on ADC map

**Fig. 5:**
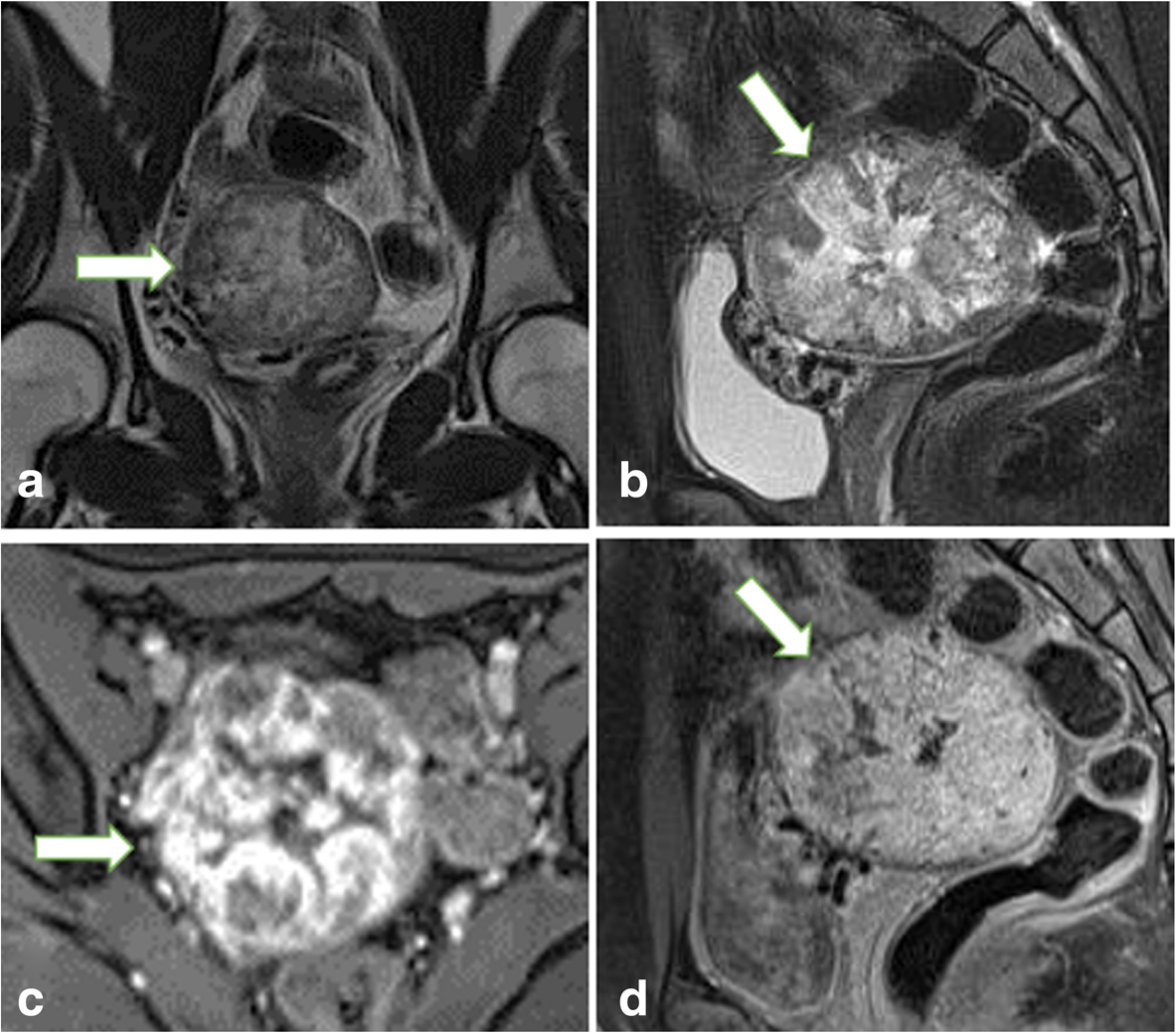
An 18-year-old woman with histological proven SST at the right ovary. On coronal T2WI (**a**) and sagittal FS-T2WI (**b**), the solid mass (arrow) appears as the “comb” sign with centrally hyperintense signal surrounded by peripherally isointense signal. After injection of the contrast medium, the mass shows flush-in on early stage enhancement (**c**) and flush-out effect on late stage postcontrast image (**d**)

**Fig. 6:**
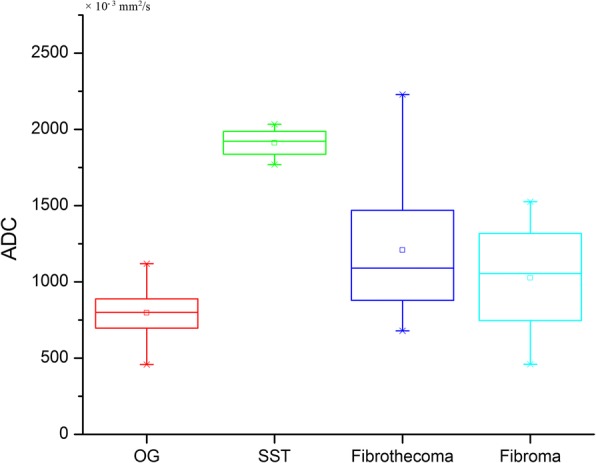
Stem-and-Leaf Plots of the calculated ADC values (10^− 3^/mm^2^/s) within four groups. The mean ADC value in OGCT is lower than that in other three groups (*p* = 0.002) with some overlap with fibrothecoma and fibroma

**Fig. 7:**
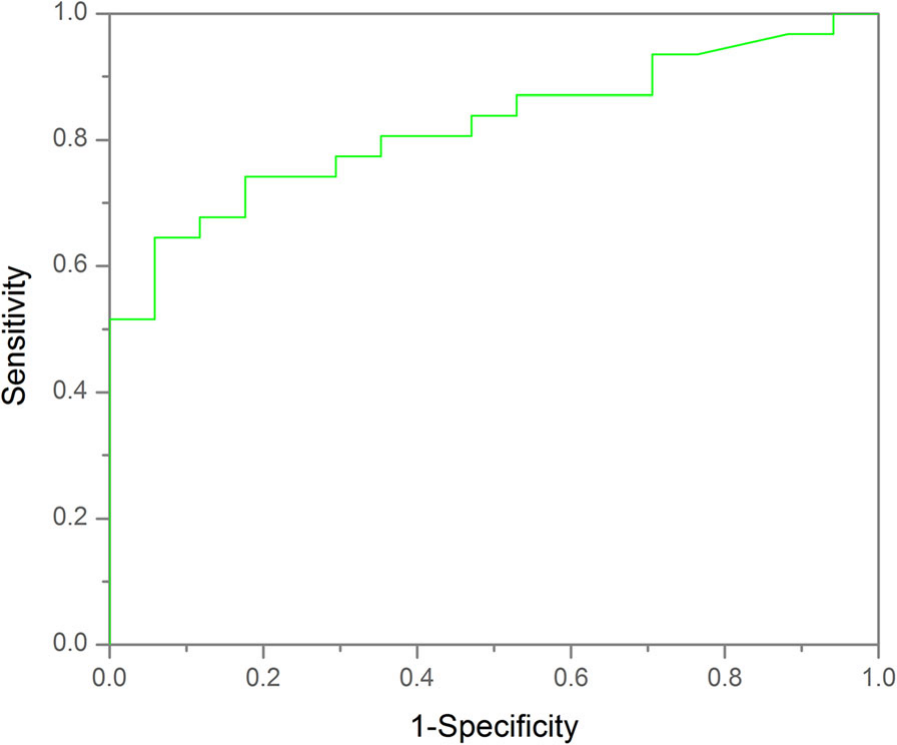
The diagnostic performance of ADC value in discriminating OGCT from OSC

Neoplastic bleeding can be seen in six OGCTs, appearing as the patchy high signal intensity on T1WI images in the tumor body (Fig. 3), which was not identified in OSC group (*p* = 0.000). Seventeen OGCTs were round or oval masses with regular margin (17/20) and intact capsule (16/20), while 33 (33/34) and 34(34/34) observed in OSCs, respectively (*p* = 0.015). Regarding the tumor component, OGCTs mostly appeared as the solid or mostly solid component (16/20, Fig. 4), while OSCs always showed purely solid component (28/34) (*p* = 0.000). On the post-contrast images, fourteen lesions (14/20) in OGCT group displayed mild enhancement and six showed moderate enhancement. In OSC group, most of fibromas showed minor enhancement and 4 SSTs appeared inhomogeneously avid enhancement (Fig. 5). On DWI images, 80 % of OGCTs (16/20, 80.0%) showed high signal intensities in comparison with 47% (16/34, 47.1%) in OCS group. The average ADC value (817 ± 144 × 10^− 3^ s/m^2^) in OGCT group was obviously less than OSC group (1223 ± 473 × 10^− 3^ s/m^2^, *p* = 0.002) and fibrothecoma (1209 ± 437 × 10^− 3^ s/m^2^, *p* = 0.001, Fig. 6). When use the ADC cutoff value as 619 × 10^− 3^ s/m^2^, MRI could yield a sensitivity of 79.4% and a specificity of 60.0% for diagnosis of OGCT, respectively; the AUC is 0.784(95% CI:0.658–0.910) (Fig. 7). Enlarged lymph nodes were not observed in all OGCTs at MRI images. Pathological fluid was only noted in one OGCT, obviously less than 11 cases in OSCs group. At multivariate analysis, neoplastic hemorrhagic contents (OR: 3.429), component (OR: 2.50) and no pathological fluid (OR: 8.130) are more indicative of OGCT diagnosis (Table 2). On MRI, two readers accurately determined the malignant condition in 17 cases. If combining ADC values, then they could yield a sensitivity of 95.0% and a specificity of 94.1% for OGCT diagnosis. Three lesions were misdiagnosed as uterine fibroids and two lesion as fibrothecoma before invasive procedures. The overall diagnostic performance of MRI for diagnosing OGCT is listed as Table 3.

**Table 3:** Diagnostic performance of MRI in diagnosis OGCTs according to two reading protocols

Protocol	SEN (%)	SPE (%)	PPV (%)	NPV (%)	ACC (%)
Conventional MRI	85.0(17/20) [63.9–95.0]	94.0(32/34) [81.0–98.0]	89.5(17/19) [68.6–97.1]	91.4(32/35) [77.6–97.0]	90.7(49/54) [80.1–95.9]
Conventional MRI plus ADC value	95.0(19/20) [76.4–99.1]	94.1%(32/34) [80.9–98.4]	90.5(19/21) [71.1–97.4]	96.9(32/33) [84.7–99.5]	94.4(51/54) [84.9–98.1]

Numbers in parentheses are the data used to calculate the percentages; Numbers in brackets are 95% confidence intervals; Conventional MRI includes T1WI, T2WI, contrast-enhanced MRI and DWI

## Discussion

OGCTs account for less than 5% of all malignant ovarian tumors, representing the most common malignant sex cord–stromal tumor in ovary origin; clinically, it may require additional chemotherapy after tumor removal surgery [13]. Radiological knowledge of this rare ovarian tumor is still limited in the reported literature, especially focusing on MR acquisition. Herein, we collected 20 OGCTs in 18 patients with prospective MR acquisition data at our single institution within nearly 10 years. To the best of our knowledge, this is the first study to describe the detailed MRI characteristics in the largest OGCT samples.

Owing to production of estrogen, OGCTs can be associated with endometrial hyperplasia, polyps, and carcinoma [18]. In our studied samples, we did observe endometrial polyps in two patients and vaginal bleeding in one patient. All other accompanied lesions were incidentally detected on MRI. In terms of tumor component, OGCTs appeared as purely solid (8/20) to entirely cystic (4/22) mass with various morphology. Our findings are in accordance with the literature that OGCTs has more heterogeneity than OSCs. It is reported that a spongelike, multilocular cystic mass filled with blood degradation products is characteristic MR imaging sign for OGCT [2, 9, 19]. We observed similar MRI appearances in 6 cases (Fig. 3). In our study, over 50% of OGCTs (11/20) displayed high or mixed signal intensity on T1WI images, which may be related with the blood degradative components. Kim et al. reported that hemorrhage in the tumor was a common MRI finding in their seven cases [20]. This feature may be useful in discriminating OGCT from OSC since it is not noticed in the latter group. Regarding the lesion enhancement, 22 tumors examined in the present study showed mild (14/22, 63.6%) and moderate (8/22, 36.4%) enhancement relative to that of the myometrium. Avid and minor enhancement were not identified in OGCTs; while 4 SSTs noticed with avid enhancement and 12 fibromas and 4 fibrothecomas with minor enhancement. Although the enhancement type between OGCT and OSC did not differ significantly, it could be useful in discriminating them from broad ligament fibroids because the latter always show marked enhancement after injection of contrast medium.

Being a problem-solving modality, MRI could provide valuable information in differentiating malignant tumors from benign gynecological diseases. Numerous studies focusing on this issue have been reported with promising results [15, 21, 22]. In current study, MRI could well indicate the morphology and components of the OGCTs with an accuracy of approximately 90% in distinguishing OGCT from OSC. Four lesions were preoperatively misdiagnosed as fibroid (three lesions) and fibrothecoma (one lesion) because of either small size or homogeneous signal intensity. Pathological fluid (a large amount of ascites) was a rare condition in OGCT group (one case); however, it more often occur in OSC group (11 cases). The true mechanism is still unknown and may result from more estrogen secretion in the latter group. No lymphadenopathy was detected on MRI in all OGCT cases, which means it is a low potential malignant tumor unlike ovarian epithelial cancer. In one recent study, the authors also concluded that lymphadenectomy was not recommended in initial staging surgery of ovarian sex cord stromal tumor due to the low lymph node metastasis rate [23].

By displaying water molecule mobility (Brownian motion), DWI is considered as a functional imaging technique, permitting the quantitative evaluation of tumor tissues with ADC value. Theoretically, as a result of high cell densities and abundant cellular membranes, the movement of water molecule in cancer tissues is restricted on DWI images and then, the derived ADC value should be generally lower in malignant disease than in benign or healthy tissue [8, 24]. Many studies have reported ADC value could help to distinguish ovarian malignant lesions from benign conditions on both 1.5 T [25–27] and 3.0 T MR system [28, 29]. In one study, the authors reported that the mean ADC value of OGCT was 1000 ± 120 × 10^− 3^ s/m^2^ in their three cases with a 1.5 T MR machine using the same *b* value [30], which is higher than our results(817 ± 144 × 10^− 3^ s/m^2^). As for MR imaging, sometimes, OGCT need to be differentiated with fibrothecoma and ligamental myoma. Our results show that the mean ADC value of OGCT is lower than OSC with statistically significant difference. The similar results is not reported in previously published studies. However, owing to the limited reported cases, the comparative ADC value should be concluded in the large cohort data. If combining the ADC value, MRI yield a sensitivity of 95.0% and a specificity of 94.1% in diagnosis of OGCT, higher than with conventional MRI reading session alone. Our findings demonstrate that there is an overlap in the ADC value between OGCT and fibroma and fibrothecoma; however, there is no overlap observed between OGCT and SST. The possible reason may be that cystic changes often occur in the large tumor in both OGCT and fibrothecoma, resulting in a wide range of measurable ADC values.

There are several limitations to this study. First, we retrieved MR reports with suggested OGCT and OSC diagnosis on PACS system (within 6 years) and then, compared the MR results with pathological reports case by case. We do believe some cases may be missed for those not mentioned on MRI reports. So, the limited study samples in both OGCT and OSC group might have influenced the final results. Second, the ADC value was manually measured on the selected area based on individual habits. Standardization in measurement may influence the final results. Third, our study is based on 1.5 T MRI system while 3.0 T MRI has been used for a decade. Owing to the variable selected *b* – value on DWI images and the limited case studies on 3.0 T, we cannot compare the ADC value between these two modalities. However, for fibrothecoma, the mean ADC value do not differ with the results reported by our previous study with 3.0 T [31]. Studies with more OGCT samples on 3.0 T unit still be needed to determine the true differences in the future.

## Conclusion

In conclusion, MRI could provide useful information in accurately diagnosing OGCT. The heterogeneous signal intensity on both T1WI and T2WI and high signal intensity on DWI images are more suggestive of OGCT diagnosis. ADC value might be useful in differentiating OGCT from OSC.

## Abbreviations

ADC: Average diffusion coefficient
CT: Computed tomography
DWI: Diffusion weighted imaging
MRI: Magnetic resonance imaging
OGCT: Ovanrian granulosa cell tumor
OSC: Other sex-cord stromal tumor
T1WI: T1 weighted imaging
T2WI: T2 weighted imaging
US: Ultrasound
